# Nivel de satisfacción de usuarios atendidos en el consultorio de odontología de un Hospital de Cajamarca, Perú 2024. Un estudio transversal

**DOI:** 10.21142/2523-2754-1303-2025-252

**Published:** 2025-08-31

**Authors:** Angel Jhosep Tapia Mantilla

**Affiliations:** 1 Universidad Nacional de Trujillo. Trujillo, Perú. angeltapiamantilla08@gmail.com Universidad Nacional de Trujillo Universidad Nacional de Trujillo Trujillo Peru angeltapiamantilla08@gmail.com

**Keywords:** satisfacción del paciente, percepción, pacientes, hospitales, cuidado de salud, patient satisfaction, perception, patients, hospitals, health services

## Abstract

**Objetivo:**

El objetivo del estudio fue establecer el nivel de satisfacción de cada uno de los pacientes atendidos en el consultorio de odontología del Hospital Regional Docente de Cajamarca en el lapso del año 2024.

**Materiales y métodos:**

Se empleó una metodología cuantitativa, descriptiva y de corte transversal, así como un diseño probabilístico aleatorio simple para escoger la muestra, que contó con 340 participantes. En cuanto a la adquisición de datos, se empleó el instrumento de medición SERVQUAL, modificado para la consulta odontológica.

**Resultados:**

En relación con la satisfacción de los usuarios atendidos en el servicio odontológico, se encontró un 39,4% de insatisfacción, un 34,4% de complacencia y un 26,2% de satisfacción.

**Conclusión:**

Los resultados revelaron un notable nivel de insatisfacción, lo que subraya la necesidad de optimizar de manera continua la calidad del servicio para mantener altos niveles de satisfacción.

## Abstract (en)

**Objective:**

The objective of the study was to establish the level of satisfaction of each of the patients seen in the dentistry office of the Cajamarca Regional Teaching Hospital during the year 2024.

**Materials and methods:**

For this purpose, a quantitative, descriptive and cross-sectional methodology was used, and a simple randomized probabilistic design was used to select the sample, with 340 participants. As for data acquisition, the SERVQUAL measurement instrument modified for the dental office was used.

**Results:**

Regarding the satisfaction of users treated in the dental service, 39.4% were dissatisfied, 34.4% were satisfied, and 26.2% were satisfied.

**Conclusion:**

The results revealed a significant level of dissatisfaction, reflected at 39.4%, underscoring the need to continuously optimize service quality to maintain high levels of satisfaction.

## INTRODUCCIÓN

Todos los seres humanos cuentan con el derecho de recibir atención médica y, al hacer uso de dicho servicio, llevan consigo expectativas con respecto a la experiencia que vivirán en las instituciones y entidades respectivas, sean públicas o privadas. La satisfacción de los usuarios está circunscrita en función de la atención y su calidad, y es importante conocer las necesidades de cada usuario para configurar dicha atención ^1^. Por ello, es importante revisar periódicamente el estado de satisfacción de los individuos para detectar puntos de debilidad y, además, espacios de mejora a nivel de organización sociocultural, técnicas de atención, entre otros aspectos esenciales ^2^. 

La OMS, en su momento, indicó que la satisfacción se encuentra vinculada al cuidado de excelencia a los beneficiarios en toda área de los servicios de salud ^3^, incluyendo la odontología. Esto cobra mayor importancia en regiones como Latinoamérica, donde existen estudios que sitúan los niveles de calidad por debajo de la media del 20% y el 30% en diversas regiones, y en sus respectivas poblaciones ^2^, lo cual se relaciona con el vínculo entre odontólogo y paciente, y otros factores evaluables ^4^.

La relevancia de este tema tiene que ver con estudios académicos que exploran la conexión entre el mundo subjetivo de los usuarios (percepciones, opiniones, expectativas y, finalmente, la sensación de contento) y la eficacia del servicio que se recibe en instituciones propias de los distintos sistemas sanitarios ^5^^-^^9^.

En el Perú, se indicó que, en términos generales, el 73,7% de los usuarios de consulta externa se sentían conformes con el servicio brindado; este estudio, sin embargo, no incluyó el servicio de odontología ^10^. Otras investigaciones indican que los componentes sobre los cuales suele estudiarse la excelencia en el servicio inciden directamente en la complacencia de cada paciente ^11^, aunque esto puede variar si la atención atenta contra sus expectativas o la capacidad de respuesta percibida ^12^.

En el contexto de la región Cajamarca, se observa una notoria escasez de estudios orientados a evaluar la satisfacción del usuario externo en los servicios que ofrecen los hospitales públicos. Hasta el momento, no se han reportado investigaciones que analicen específicamente las percepciones de los pacientes sobre la atención en el área de odontología. Esta falta de evidencia científica justifica la necesidad de desarrollar el presente estudio, el cual se plantea como una contribución pionera que permitirá generar información relevante para mejorar la calidad del servicio odontológico y orientar futuras estrategias de gestión en salud.

En ese sentido, este artículo busca unirse a los esfuerzos de los estudios aludidos anteriormente desde un lugar en específico: el Hospital Regional Docente de Cajamarca (HRDC) nivel II-2, donde la alta demanda de pacientes que buscan atención en la consulta externa odontológica hace necesario un análisis de la situación desde el enfoque del usuario.

## MATERIALES Y MÉTODOS

El presente estudio fue aprobado por el Comité de Ética e Investigación del HRDC, mediante Carta N.^o^ 146-2024-GR.CAJ/DRS/HRDC/CDEI.

Este trabajo fue de tipología cuantitativa, dado que se obtuvieron y analizaron resultados expresados de manera numérica para analizar los fenómenos abordados y obtener conclusiones sólidas y objetivas. Fue, además, un estudio transversal por cuanto el acopio de información se realizó en un determinado tiempo en la consulta.

### Diseño de estudio y muestra

La muestra fue escogida mediante un diseño probabilístico aleatorio simple y se calculó un total de 340 personas mediante fórmula para poblaciones finitas.

En la formula, se tomó en cuenta para el valor de *n* los 2944 pacientes asegurados, mayores de edad, que recibieron atención en 2023, en el consultorio de odontología del HRDC.


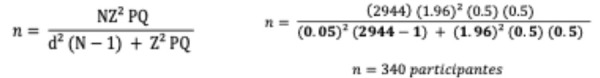


### Instrumento

Para la recolección de datos, se usó el instrumento de medición SERVQUAL ^10^, modificado para la consulta odontológica, y cuya validez fue confirmada mediante juicio de expertos. 

El instrumento de medición utilizado en este estudio evalúa cinco dimensiones fundamentales: confiabilidad, capacidad resolutiva, bioseguridad, empatía y elementos tangibles. Está estructurado en dos cuestionarios compuestos por 22 ítems cada uno. El primero de ellos se orienta a la medición de las expectativas del usuario en relación con el servicio que va a recibir, mientras que el segundo se enfoca en las percepciones, es decir, en la experiencia real proporcionada. Ambos instrumentos emplean una escala de valoración de 7 puntos, la cual se categoriza en tres niveles: satisfacción (puntuaciones de 6 y 7), complacencia (puntuaciones de 4 y 5) e insatisfacción (puntuaciones de 1 a 3).

Se obtiene el nivel de satisfacción del cuestionario de percepciones, ya que evalúa el servicio brindado.

### Análisis estadístico

Los datos recogidos fueron ingresados en una hoja de Excel y, posteriormente, fueron transferidos al programa SPSSv.26. Finalmente, fueron presentados de manera clara mediante tablas.

## RESULTADOS

Se contó con la participaron de 340 usuarios mayores de edad, atendidos en el consultorio de odontología del HRDC. 

En la Tabla 1 se presentan los resultados de la satisfacción en usuarios que evalúan la atención general en el consultorio odontológico, donde el 39,4% de los usuarios manifestó insatisfacción en sus percepciones, lo cual representa el nivel más alto de insatisfacción reportado. 

**Tabla 1:** Satisfacción de usuarios en consultorio odontológico del HRDC

	Expectativas		Percepciones		P
Niveles	n	%	n	%	
Satisfacción	104	30,6%	89	26,2%	0,001
Complacencia	114	33,5%	117	34,4%	
Insatisfacción	122	35,9%	134	39,4%	
Total	340	100,0%	340	100,0%	

En las tablas siguientes se presentan los resultados con base en las dimensiones del instrumento. 

En la Tabla 2 se hace referencia a las expectativas de los usuarios en la dimensión de confiabilidad, donde hay un nivel de complacencia con un 40,3% en percepciones. En cuanto a la tabla 3, expone los resultados acerca de la capacidad resolutiva, con un 46,2% que evidenció complacencia en las percepciones. La Tabla 4, que contiene los resultados referentes a la bioseguridad, demuestra satisfacción, con un 49,4% en percepciones. Con relación a la Tabla 5, referente a la empatía, se observan niveles de complacencia con un 52,9% en percepciones. Finalmente, en la Tabla 6, la cual aborda los resultados de los elementos tangibles, se evidencia insatisfacción con un 37,6% en percepciones.

**Tabla 2:** Satisfacción de usuarios, con respecto a la confiabilidad

	Expectativas		Percepciones		P
Niveles	n	%	n	%	
Satisfacción	119	35,0%	118	34,7%	0,028
Complacencia	124	36,5%	137	40,3%	
Insatisfacción	97	28,5%	85	25,0%	
Total	340	100,0%	340	100,0%	

**Tabla 3:** Satisfacción de usuarios, con relación a la capacidad resolutiva

	Expectativas		Percepciones		P
Niveles	n	%	n	%	
Satisfacción	147	43,2%	111	32,6%	0,037
Complacencia	84	24,7%	157	46,2%	
Insatisfacción	109	32,1%	72	21,2%	
Total	340	100,0%	340	100,0%	

**Tabla 4:** Satisfacción de usuarios, frente a bioseguridad

	Expectativas		Percepciones		P
Niveles	n	%	n	%	
Satisfacción	213	62,6%	168	49,4%	>0,001
Complacencia	69	20,3%	92	27,1%	
Insatisfacción	58	17,1%	80	23,5%	
Total	340	100%	340	100%	

**Tabla 5:** Satisfacción de usuarios, referente a la empatía

	Expectativas		Percepciones		P
Niveles	n	%	n	%	
Satisfacción	131	38,5%	86	25,3%	0,001
Complacencia	127	37,4%	180	52,9%	
Insatisfacción	82	24,1%	74	21,8%	
Total	340	100%	340	100%	

**Tabla 6:** Satisfacción de usuarios con relación a elementos tangibles

	Expectativas		Percepciones		P
Niveles	n	%	n	%	
Satisfacción	91	26,8%	88	25,9%	0,001
Complacencia	118	34,7%	124	36,5%	
Insatisfacción	131	38,5%	128	37,6%	
Total	340	100%	340	100%	

## DISCUSIÓN

El presente estudio consistió en determinar el nivel de satisfacción de los usuarios atendidos en el consultorio odontológico. Los hallazgos indican que las diferencias entre los porcentajes son significativamente relevantes, lo que muestra un nivel de insatisfacción en el usuario. 

En ese sentido, los resultados se relacionan con lo reportado por Guevara en el HRDC, donde reveló que el 61,85% de los pacientes manifestaron insatisfacción con la calidad de los servicios recibidos, utilizando el instrumento SERVQUAL ^13^. De manera complementaria, Bustamante y Gálvez, en su estudio efectuado en el mismo establecimiento hospitalario en Cajamarca, identificaron que el 46% de los usuarios expresaron descontento respecto de la atención recibida ^14^. Asimismo, Palmieri, en su tratado, desarrollado con pacientes odontológicos de Córdoba (Argentina), reveló un alto nivel de insatisfacción del 86% ^15^. 

Por el contrario, estos resultados difieren de lo reportado por Aguilar, en Loja (Ecuador), donde el 49,73% de los pacientes manifestó satisfacción con el servicio recibido ^16^. De igual modo, Becerra, en un centro de salud de Cajamarca, evidenció niveles generales de satisfacción; esto puede deberse a las variaciones en los enfoques metodológicos y las muestras examinadas ^17^.

En cuanto a la dimensión de confiabilidad, lo resultados coinciden con Gonzales, Álvarez y Chaparro, quienes en Ixtlahuaca, México, identificaron falencias en esta dimensión tras la aplicación del modelo SERVQUAL para evaluar la percepción de calidad en los servicios odontológicos. Se identificaron debilidades específicas en esta dimensión, lo cual indica que es necesario mejorar este aspecto para optimar la experiencia del usuario ^18^. Por otro lado, no coinciden los hallazgos del estudio con Baltazar, en Cerro de Pasco, que reportó una insatisfacción aun mayor ^19^; mientras que Montoya, en Medellín, llegó a resultados discrepantes al descubrir una alta satisfacción en la confianza depositada en los servicios dentales, lo que contribuye positivamente a la salud bucal del paciente ^20^.

Respecto de la capacidad resolutiva del servicio, presenta similitudes con el trabajo de Mazzeo *et al*., en La Plata (Argentina), quienes revelaron un nivel considerable de satisfacción ^21^. En consonancia, Condezo y Caldas, en Lima, también identificaron un 68,92% de satisfacción ^22^. No obstante, lo encontrado en este trabajo se ubica por debajo de lo hallado por Leveau y Merino en Iquitos, con un 83,5% (^23^), y por Bustamante y Cabrera en Cuenca (Ecuador), donde el 93,5% de los usuarios indicaron satisfacción ^24^. Estas discrepancias pueden atribuirse a factores contextuales propios de cada institución de salud. 

En lo que respecta a la bioseguridad, los resultados guardan similitud con los de Enciso y Lizarbe en Chiclayo, quienes hallaron que el 42% de los usuarios experimentaban satisfacción ^25^. En contraste, Espino, en Chasquipampa, no encontró variaciones sustanciales entre expectativas y satisfacción ^26^. Por otro lado, Rodríguez, en Recuay, expresó que la insatisfacción en bioseguridad se relacionaba con la ausencia de mejoras sustanciales ^27^.

Con relación a la empatía, los resultados concuerdan con los de Sharka *et al*., en Taiwán, donde se identificaron deficiencias en comunicación y empatía del personal odontológico, lo cual sugiere la necesidad de fortalecer las competencias interpersonales para mejorar la confianza y la satisfacción del paciente ^28^. Por el contrario, Wong, en Chiclayo, reportó una percepción elevada de empatía, lo que contrasta con los resultados obtenidos en este análisis ^29^.

Finalmente, en cuanto a los elementos tangibles, los resultados difieren con los obtenidos por Iza *et al*., en Ecuador, donde encontraron altos niveles de satisfacción, a pesar de requerirse mejoras generales ^30^. De manera similar, Becerra *et al*., en Ica, identificaron altos niveles de satisfacción general, incluyendo esta dimensión ^31^. En contraposición, Febres y Mercado, en Huancayo, reportaron una insatisfacción del 57,1% en elementos tangibles ^32^.

Entre las fortalezas de este estudio destaca el acceso directo a la población usuaria, lo que permitió aplicar el cuestionario de manera personalizada. Asimismo, la investigación utilizó un instrumento estructurado y validado para la medición del nivel de satisfacción, lo que otorga consistencia a los resultados obtenidos. También resalta la aplicabilidad práctica de los resultados, ya que estos pueden servir como una herramienta de gestión para las autoridades del hospital, al identificar puntos críticos que requieren atención o mejora.

Se encontró como principal limitación el sesgo en el cuestionario llenado por los usuarios que recibieron atención en el consultorio odontológico. Esto implica que las respuestas proporcionadas por los pacientes podrían no representar con precisión la calidad real del servicio ofrecido. Es fundamental que toda institución implemente procesos de evaluación continua, con el fin de optimizar sus servicios y responder adecuadamente a las necesidades de los usuarios.

Se recomienda establecer mecanismos de comunicación que permitan alinear las expectativas de los pacientes con las condiciones reales del servicio, fomentar la capacitación continua del personal y llevar a cabo evaluaciones periódicas de satisfacción, con el objetivo de perfeccionar los servicios conforme a las necesidades y sugerencias expresadas por los usuarios.

## CONCLUSIÓN

Este artículo permitió concluir que los usuarios que recurrieron al HRDC por atención en materia de odontología evidenciaron una notable insatisfacción. Según las dimensiones evaluadas, se obtuvo niveles de complacencia en confiabilidad, capacidad resolutiva y empatía; satisfacción en la dimensión de bioseguridad, e insatisfacción en los elementos tangibles. Por tanto, si bien existen áreas con niveles de complacencia o satisfacción, la percepción general aún tiende a la insatisfacción.

## Financiamiento:

Ninguno
